# Ultrasound and heat treatment effects on *Staphylococcus aureus* cell viability in orange juice

**DOI:** 10.1016/j.ultsonch.2021.105743

**Published:** 2021-09-07

**Authors:** Akila Amir Tahi, Sérgio Sousa, Khodir Madani, Cristina L.M. Silva, Fátima A. Miller

**Affiliations:** a Laboratoire de Biomathématiques, Biophysique, Biochimie, et Scientométrie (L3BS), Faculté des Sciences de la Nature et de la Vie, Université de Bejaia, 06000 Bejaia, Algeria; b Universidade Católica Portuguesa, CBQF – Centro de Biotecnologia e Química Fina – Laboratório Associado, Escola Superior de Biotecnologia, Rua Diogo Botelho 1327, 4169-005 Porto, Portugal; c Centre National de Recherche en Technologie Agro-alimentaire, Université de Bejaia, 06000 Bejaia, Algeria

**Keywords:** *Staphylococcus aureus*, Ultrasound, Heat treatment, Thermosonication, Kinetic inactivation modelling, Cell damage

## Abstract

Ultrasounds are being considered an excellent alternative technology in juice preservation. Yet, when combined with heat treatment, the process seems to be further intensified. This work aimed to evaluate and compare the impact of ultrasounds and heat treatments, when applied alone or in combination, on *Staphylococcus aureus* survival in orange juice. Inoculated commercial pasteurized orange juice was treated at different times at 20, 30, 40, 50 and 60 °C. SEM analyses were applied to identify morphological changes in *S. aureus* cells appearance. The microbial inactivation data were fitted using two mathematical models, depending on the behaviour observed.

Sonication at 20, 30, and 40 °C induced 4.02 ± 0.52, 3.80 ± 0.49 and 4.30 ± 0.74 log cycles reduction of *S. aureus* after treatments of 90, 60 and 60 min, respectively. The heat treatments at the same temperatures had no impact on *S. aureus* survival. When 50 and 60 °C were applied, more than 5-log reductions were attained for both thermosonication and heat treatments alone. A synergistic effect was observed between sonication and high temperatures. At 50 °C, the thermosonication reduced the treatment time from 60 to 35 min and the microbial load from 5.14 ± 0.08 to 10.76 ± 0.28 log cycles reduction, compared to heat treatment alone. Results from SEM images showed that cells undergo membrane damage during sonication exposure. This was observed by collapsed cells, cell disruption, and holes in the cell’s membrane.

Thermosonication proved to be a viable alternative to thermal pasteurization of orange juice since milder treatments can be safely applied, improving the final product quality.

## Highlights

• Thermosonication (TS) was more effective than sonication and heat treatment alone.
• Ultrasounds reduced the bacterial counts by 4 log cycles at the end of treatments.
• TS leads to 5-log reductions at 50 and 60 °C after 1 and 25 min, respectively.
• Weibull model was adequate to describe the majority of the inactivation behaviour.
• Morphological changes were observed in *S. aureus* TS-treated cells by SEM images.

## Graphical abstract

**Figure:**
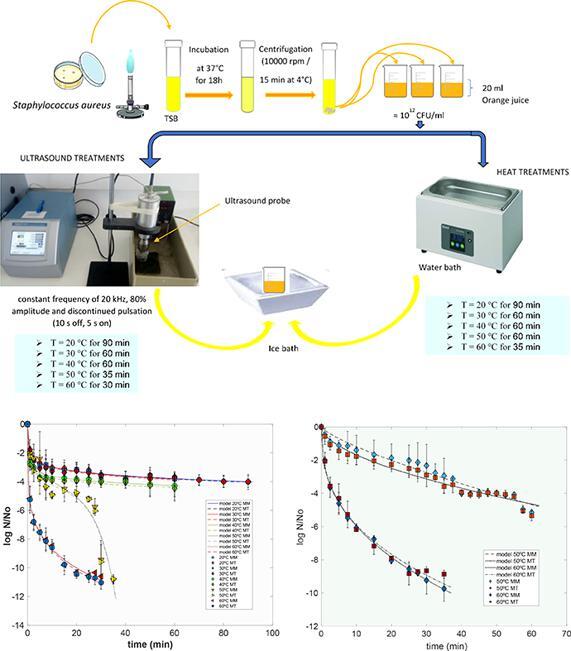

## 1 Introduction

Foodborne diseases (FBD) are a primary concern. The exact impacts of unhealthy food on well-being and the total economic loss are unknown, but the global effect on health, commerce and prosperity is believed as massive [1].

Due to systematic underreporting and difficulties in attributing diseases with numerous transmitting mechanisms to food intake, the determination of the actual number of FBD is a challenge. Despite this, substantial progress has been made in recognizing the FBD load, with robust figures from many developing countries indicating that a large proportion of the population (12–33 %) is affected each year. Many of these multi pathogen disease estimates have shown a high role of *Staphylococcus aureus* [2], [3].

*S. aureus* is an aero-anaerobic facultative, nonsporulating, nonmotile bacterium, gram-positive and catalase-positive, which presents as a cluster shell (bunches of grapes). The *S. aureus* species is commensal to man, and it is a ubiquitous germ that can be found in the environment, air, dust, sewage, water, environmental surfaces, humans and animals [4], [5]. It is a halophilic bacterium (tolerates high salt concentrations) and can also grow in a wide range of temperatures and pH [6].

*S. aureus* is an opportunistic pathogen in some locations or under some circumstances. Its pathogenicity is related to several specific secretions, such as enzymes (coagulase) and toxins (endotoxins) [4]. Studies conducted by Bhatia & Zahoor [4] confirmed that a minimum of 10^5^ *S. aureus* cells in one gram of food are needed to secret a sufficient amount of enterotoxin to cause disease.

*S. aureus* is among the most significant pathogens causing a broad spectrum of diseases in humans and animals. The standing of orange juices as the main vehicles of human foodborne diseases poses a significant challenge for sectorial coordination control efforts within the fruit juice industry. Therefore, the need to develop and validate effective inactivation processes becomes extremely important. Since the objective is to meet the increasing consumer demand for safe and quality food, non-thermal methods are increasingly being explored. One of these preservation technologies is sonication.

Ultrasound is considered an efficient process as it fulfills the Food and Drug Administration requirements for a 5-log reduction of microbial cells in fruit juices [7], [8]. Its “non-thermal” nature also minimizes the adverse effects of heat on the organoleptic and nutritional characteristics of the food product [9]. Besides all these features, it is considered a “green” technology because it involves healthy energy and does not require any additional chemicals or, in some exceptions, a minimal quantity. Ultrasound uses physical and chemical phenomena that are fundamentally different from those applied in conventional extraction, processing or preservation techniques [10].

Sonication is ultrasound waves with frequencies above the human hearing range (>16 kHz). In liquid environments induces acoustic cavitation, which is the creation, development and subsequent collapse of bubbles. When ultrasound waves disperse, the bubbles oscillate and collapse, causing thermal, mechanical and chemical effects [11], [12]. The physical impacts caused by the breakdown of the acoustic bubble include extreme shock waves and shear. This form of mechanical effect is known to weaken and damage biological cell membranes. Therefore, this method has been currently used to release cell material in microbiology laboratories [[13], [14]]. The chemical effects of cavitation bubble collapse are the formation of free radicals HO^•^, O^•^ and H^•^ resulting from the decomposition of water [15]. The collapse of the cavitation bubble also leads to the liberation of a high temperature of 5000°K and a high pressure equivalent to thousands of atmospheres [11].

A combination of sonication and heat has been used in a method called thermosonication (TS). This process has been increasing the microbial inactivation rate, in which a synergistic effect is being observed[[16], [17], [18], [19]]. However, there is a higher temperature limit for microbial inactivation with TS. Due to an amortization effect, the bubbles filled with steam will generate less powerful implosion, leading to a decrease in the temperature and pressure released at elevated temperatures. This phenomenon results in similar inactivation rates of TS treatment using higher temperatures and a thermal process alone [20].

Most ultrasound and thermosonication studies continue to assume the microbial inactivation behaviour as first-order kinetics. In terms of process design purposes, this may result in unsafe treatments. This is especially important in thermosonication works, where there is evidence that the microbial behaviour can be sigmoidal[21].

The main objective of this study was to assess the *S. aureus*’s kinetic inactivation behaviour in orange juice at different temperatures and after ultrasound, thermosonication and heat treatments. The Weibull and a four-parameter model were used to describe the microbial inactivation behaviour under these processes. *S. aureus* cell viability and damage were also evaluated using two different platting media and SEM analyses.

## 2 Materials and methods

### 2.1 Bacterial strain and inoculum preparation

A loop of a bacterial colony from *S. aureus* ATCC 29213 was transferred into 5 ml of Tryptic Soy Broth (TSB, Biokar diagnostics, France) and incubated at 37 °C overnight to prepare a final working stock bacterial culture.

4 ml of the working culture was then added into 100 ml of fresh TSB and incubated in a shaking bath at 37 °C to obtain a final bacterial suspension. For this last step, the incubation period was set so that the bacteria culture was at the exponential phase (log phase).

The microorganism growth curve was recorded by measuring the change in the optical density of the culture suspension over time, at a wavelength of 600 nm, using a spectrophotometer (Novaspec II Visible Spectrophotometer).

Cells at the exponential phase were harvest by centrifugation at 10,000 g for 10 min at 4 °C (Hettich Rotina 380R centrifuge; Germany) and washed three times by resuspension in buffered peptone water (Biokar diagnostics, France).

Optical density measurements followed by plate counting were used to prepare bacteria suspensions with an initial concentration of 10^12^ CFU/ml.

### 2.2 Orange juice

A commercial pasteurized orange juice (100% orange juice, Compal) was used in the experiments. Each orange juice sample was artificially inoculated with the prepared *S. aureus* suspension to obtain an inoculated juice with a final concentration of approximately 10^12^ CFU/mL.

### 2.3 Treatments

#### 2.3.1 Ultrasound treatments

20 ml of orange juice containing 10^12^ CFU/ml of *S. aureus* were placed in a 50 ml glass beaker closed with parafilm and ultrasonicated by ultrasound homogenizer (Qsonica Sonicator ultrasonic processor Part No. Q700, USA) at a constant frequency of 20 kHz, 80% amplitude and discontinued pulsation (10 s off, 5 s on). The energy input was regulated by fixing the amplitude of the sonicator probe.

**Fig. 1:**
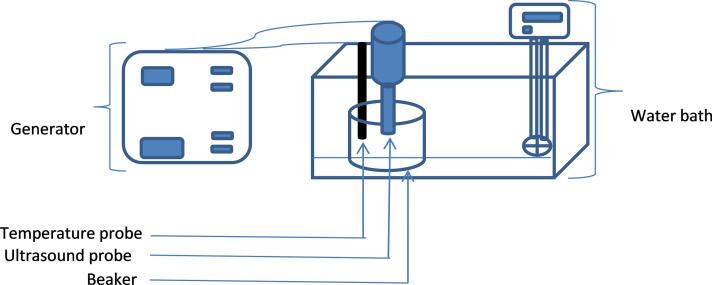
Diagram of the thermosonication treatment equipment setup.

The ultrasonic processor is coupled with a water bath to maintain a constant temperature (Fig. 1). Three temperature levels were studied: 20 °C, 30 °C and 40 °C ± 2 °C.

The processing tip (diameter 13 mm) of the ultrasonic horn was always positioned at 1.5 cm below the surface of the orange juice. In each experiment, the temperature was monitored by a temperature probe inserted inside the fruit juice beaker. An ice-water bath surrounding the juice samples ensured to keep the solution temperatures at the required values, although the heat derived from the ultrasound. Several samples were taken at different time intervals during treatments depending on the temperature chosen (seventeen sampling points were collected from 0 to 90 min at 20 °C and fourteen samples from 0 to 60 min at 30 and 40 °C). After ultrasound treatments, the samples were rapidly cooled to room temperature in an ice-water bath.

Three replicates of all these experiments were performed.

#### 2.3.2 Thermosonication treatments

The thermosonication was carried out as for the ultrasonic treatment, but the temperatures were kept at 50 and 60 °C ± 2 °C to study the synergy between temperature and ultrasounds. Thirteen sampling points were taken for each temperature at different processing times, up to a maximum of 35 min.

Three replicates of all these experiments were performed.

#### 2.3.3 Heat treatments

20 ml of orange juice were placed into a sterile glass beaker, closed with parafilm, set in a heat-controlled water bath with constant stirring and gradually heated to the desired processing temperature (20, 30, 40, 50 and 60 °C). The sample temperature was monitored with a thermometer placed into the orange juice. Once the orange juice reached the desired temperature, it was inoculated with 1 ml of the bacterial suspension.

Samples were collected at the beginning and end of treatment (90, 60 and 60 min) for low temperatures (20, 30 and 40 °C). Several sampling points were collected for high temperatures (50 and 60 °C): from 0 to 60 min at 50 °C and from 0 to 35 min at 60 °C. After that, samples were immediately cooled in an ice-water bath to stop thermal inactivation.

Three replicates of all these experiments were performed.

### 2.4 Enumeration of bacteria

Serial dilutions were performed in buffered peptone water with orange juice taken before and after treatments to assess *S. aureus* microbial load. Stress-induced sublethal cells present on juice samples were determined by spread plating the diluted samples onto two different enumeration media: Tryptone Soy Agar (TSA, Lab M, United Kingdom), which is a non-selective medium used to grow both injured and non-injured cells, and Mannitol Salt Agar (MSA, Biokar diagnostics, France) which is a *S. aureus* selective medium where only non-damaged bacteria can grow. The plates were incubated at 37 °C for 24 h.

For each experiment, all microbiological analyses were performed in triplicate.

Colony Forming Units per millilitre (CFU/ml) of bacterial suspension are estimated using the Agar Plate Cultural Colony Count Method.

The orange juice sample was analyzed for total bacterial count (TBC). The TBC was determined by the plating technique using standard methods on TSA and selective medium MSA for *S. aureus*. Results indicated that *S. aureus* and other microorganisms were not detected in the tested samples.

### 2.5 Scanning electron microscopy

Juice samples (containing the bacteria) were filtered through 0.45 µm cellulose acetate filters (Sartorius Stedim Biotech, Germany), and *S. aureus* cells were collected (together with the pulp) to be fixed and dehydrated. Fixation was performed by immersing the filters in a 2.5% (v/v) glutaraldehyde (Sigma-Aldrich) solution for at least 1 h. Next, glutaraldehyde solution was removed, and filters were washed with deionized water. Then, they were dehydrated by a graded ethanol series (30, 50, 70, 80, 90 and 100% (v/v)) through its immersion in each solution for 10 min. After removing the 100% ethanol solution, a couple of drops of hexamethyldisilazane (HMDS; Sigma-Aldrich, United States) were placed over the filters and immediately evaporated with a stream of nitrogen. Sections of the dried filters were cut and set on observation stubs, covered with a double-sided adhesive carbon tape (NEM tape; Nisshin, Japan), and coated with gold/palladium. The observation was performed in a JEOL JSM-5600LV (JEOL, Japan), with an acceleration voltage of 30 kV.

The best inactivation conditions with thermosonication (60 min at 40 °C, 35 min at 50 °C and 30 min at 60 °C) and their respective controls (untreated cells) were chosen to be studied by scanning electron microscopy (SEM) to allow the evaluation of the impact of the treatments on the *S. aureus* morphology and cells integrity.

### 2.6 Modeling and statistical analysis

The effects of treatments on *S. aureus* loads were analysed by ANOVA using SPSS Statistics 27 software (IBM Co., USA) and expressed as mean value ± standard deviation. Significance was established at P < 0.05.

Log-survival data of *S. aureus* obtained in ultrasonicated, thermal and thermosonicated treatments were fitted with the Weibull model, as shown in Equation (1):

(1) $$log\left(\frac{N}{N_{0}}\right)=-{k\times t}^{n}$$

where N is the microbial load (CFU/mL) at a given treatment time t (min), N_0_ the juice initial microbial load (CFU/mL), k is a rate parameter (min^-n^), and n the shape factor (dimensionless) indicator of upward (n < 1) or downward concavity (n greater than 1).

For thermosonicated samples at 50 °C, a four-parameter / sigmoid mathematical model (Equation (2) [21]was used to describe the microbial behaviour:

(2) $$log\left(\frac{N}{N_{0}}\right)=-{k_{1}\times t}^{n_{1}}-{k_{2}\times t}^{n_{2}}$$

where k_1_, k_2_, n_1_ and n_2_ are model parameters.

Regression analyses were done using IBM SPSS Statistics 27 for Windows® (SPSS Inc., Chicago, USA). Model adequacy was assessed by residual analysis (randomness, normality and variance of residuals) and coefficient of determination, R^2^. The precision of the model parameter estimates was evaluated by calculating confidence intervals at 95%.

## 3 Results and discussion

### 3.1 Growth curve

**Fig. 2:**
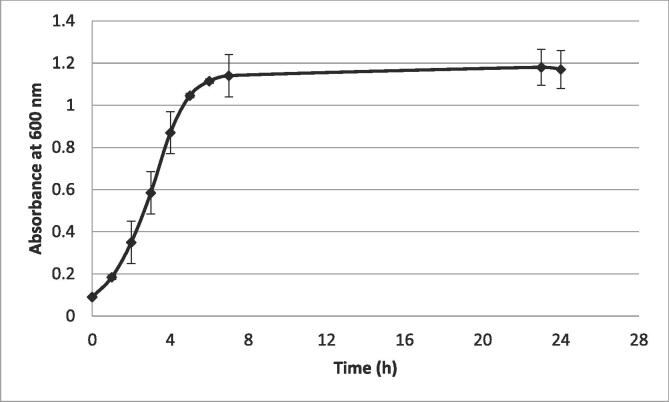
Growth curve of *Staphylococcus aureus*.

Bacterial growth is the systematic increase of all bacterium components. The growth curve has four phases: a latency, an exponential, a stationary, and a decline phase [[22], [23]]. *S. aureus* growth curve was recorded by monitoring the changes in absorbance over time (Fig. 2). As can be observed, *S. aureus* suspension reached the mid-exponential phase after 3 h of incubation. In this phase, the growth rate reaches a maximum (μ = max), and the cell mass is represented by viable cells (zero mortality)[22]. The stationary phase was obtained after 7 h of incubation, where the growth rate becomes zero (μ = 0), and the population number remains constant. The results found in this study are similar to those reported for *Staphylococcus* epidermidis by Gao, Lewis, Ashokkumar, and Hemar [24].

The latency phase (zero growth rate (μ = 0)) is the time required for the bacteria to synthesize the enzymes needed for the new substrate and depends on the bacteria’s age and the growth medium composition. If transferred to an identical medium, the bacterium has no latency phase)[23]. Based on this definition, we can understand the behaviour of *S. aureus* in this experiment. The absence of the latency phase is due to using the same culture medium during the revivification and the realization of the growth curve.

Belay & Rasooly[25] studied the growth of *S. aureus* and Staphylococcal enterotoxin A (SEA) production in aerobic and anaerobic conditions and concluded that the culture conditions influence the generation time of the bacteria. They found that the generation time for anaerobic and aerobic conditions to mid-log phase was 80 min and 35 min, respectively. They also found that the secretion of SEA depends on the bacterial growth, but the detection of SEA was after 120 min of incubation in both conditions[25].

### 3.2 Effect of ultrasound treatment on *S. aureus* inactivation behavior

This study aimed to understand if the inactivation of *S. aureus* by ultrasound treatments depends on temperature and treatment time. According to previous works, studies performed in the exponential phase are of utmost importance since this is the period of bacterial replication, where the microbial population are more vulnerable to harmful factors, such as radiation and antimicrobial drugs [[26], [27]]. Besides that, *S. aureus* ATCC 29213 can secrete staphylococcal enterotoxins SEA, SEC, and SEE at this growth phase [28]. Therefore, and based on the growth curve obtained (Fig. 2), suspensions of *S. aureus* collected at their exponential phase were prepared for ultrasound treatments.

**Fig. 3:**
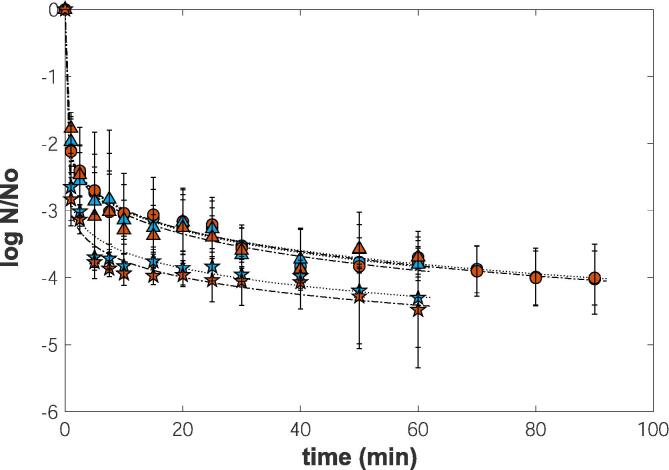
Inactivation of *S. aureus* during ultrasound treatment in orange juice at 20 °C (○), 30 °C (∆), and 40 °C (☆) in two media (Blue: MSA; Red: TSA). The lines … and ._._. represent corresponding Weibull model fit to the experimental data. (For interpretation of the references to colour in this figure legend, the reader is referred to the web version of this article.)

The kinetic inactivation behaviour of *S. aureus* treated with ultrasounds at sublethal temperatures (20, 30 and 40 °C) for different processing times was determined by plating onto selective media (MSA) and non-selective media (TSA) and can be visualized in Fig. 3.

The initial concentration of *S. aureus* was 10^12^ CFU/mL for all tested conditions. As can be observed, the ultrasound treatment reduced the viable bacterium counts by 4.02 ± 0.52, 3.80 ± 0.49 and 4.30 ± 0.74 log cycles at the end of treatments at 20, 30 and 40 °C, respectively.

Another feature that can be noted is the overall shape of the survival curves of *S. aureus* subjected to ultrasound treatments at sublethal temperatures. All the curves don’t exhibit shoulder or lag phases. They start directly with a log-linear period of maximum inactivation rate, followed by a considerable tail phase. This kinetic behaviour revealed that initially, *S. aureus* was sensitive to the ultrasound treatment, but after some time, the microbial population started to get used to and create some resistance.

To determine if the sublethal temperatures alone had the same inactivation effect on *S. aureus*, the same procedure was realized without the ultrasound treatment. These results are included in Table 1. Values showed that sublethal temperatures did not affect *S. aureus* survival. Indeed, at 20 °C, the microbial number was statistically similar before and after 90 min of heat processing. At 30 and 40 °C, an increase in bacteria number was detected after 60 min of treatment, but that increase was only significant for the selective medium at 40 °C. This behavior is explained by the expected growth of most *Staphylococcus* strains between 18 and 40 °C[29]and the proximity to the optimal growth temperature.

**Table 1:** *S. aureus* counts (CFU/mL) on non-selective and selective medium after heat treatment. The values are mean ± standard deviation.

Time(min)	20 °C		30 °C		40 °C	
	TSA × 10^-12^	MRS × 10^-12^	TSA × 10^-12^	MRS × 10^-12^	TSA × 10^-12^	MRS × 10^-12^
0	3.76 ± 1.26^a^	3.97 ± 1.70^a^	4.63 ± 0.76^a^	4.20 ± 0.87^a^	4.63 ± 1.08^a^	4.52 ± 1.00^a^
60	–	–	36.3 ± 32.1^ab^	31.7 ± 29.7^ab^	38.1 ± 30.6^ab^	64.7 ± 33.6^b^
90	6.00 ± 2.69^a^	5.93 ± 3.22^a^	–	–	–	–

For both treatments and all the temperatures tested, two plating media were employed. The use of two distinct types of media (selective and non-selective) had the goal of recover damaged and intact cells. Undamaged and damaged cells have been able to develop on non-selective media (TSA). In contrast, injured bacteria have been unable to grow on selective media enriched with sodium chloride (MSA) due to changes in membrane permeability[30].

The analysis of the results showed that there were no significant differences between counts on non-selective and selective media, regardless of temperature and treatment time. Several studies reported the detection of only viable and dead cells when ultrasound (20 kHz) processes were applied[[9], [31], [32], [33]]. They assign to the phenomenon the expression “all or nothing”. This resistance is believed to be due to the physically hard-wearing envelopes[34], to the cell wall peptidoglycan and teichoic acid content and the *S. aureus* capsule or pseudo-capsule presence [29].

The bactericidal effect of ultrasound waves is dependent on many factors, such as the thickness of the bacterial wall, the bacterial population in the area of application, and the time, frequency and intensity of the ultrasonic treatment [[13], [14], [15]]. In this study, we used a fixed frequency of 20 kHz. According to Evelyn and F. V. M. [35], high power ultrasound is acoustic waves characterized by a low frequency of 20–100 kHz and intensity of 10–1000 w/cm^2^.

Sonication was considered an effective decontaminated process by the Food and Drug Administration for fruit juices since it meets the requirements of a 5-log reduction on microbial cells [7], [8]). However, this was not the case for the sublethal temperatures tested, where the maximum inactivation of *S. aureus* achieved was 4.30 ± 0.74 log cycles at the end of ultrasonic treatment at 40 °C. Indeed, several authors already mentioned an ineffective microbial inactivation with ultrasounds when this treatment is applied at sublethal temperatures [[36], [37]]. Thermosonication showed no significant advantage on bacteria elimination when nonlethal temperature was combined with ultrasound, being the effect equivalent to ultrasonic treatment alone [12].

It is believed that the ineffectiveness of ultrasonic processes might be attributed to distinct physical and biological properties of *S. aureus* [31]. According to the literature, the cavitation bubbles generated by sonication are hydrophobic, making it easier to act on hydrophobic surfaces[24]. Unfortunately, the surface of *S. aureus* is conversely hydrophilic, leading to the cavitation bubbles collapse far away from the bacteria surface and the result slight microbial damage. The thicker cell walls present in Gram-positive and coccus-shaped *S. aureus* bacteria have also been reported to be resistant to ultrasound treatment [38]. The capsule is also a protective element that could prevent cavitation bubbles from collapsing near the plasma membrane, thus protecting the bacterial cell from disruption. It is also found that the high hydration of the capsule could play a crucial role in protection by absorbing the mechanical forces exerted on the bacterial cell[24].

### 3.3 Effect of thermosonication and heat treatment on *S. aureus* behavior

Since ultrasounds combined with sublethal temperatures did not result in a marked decrease in the *S. aureus* population, higher temperatures were applied.

**Fig. 4:**
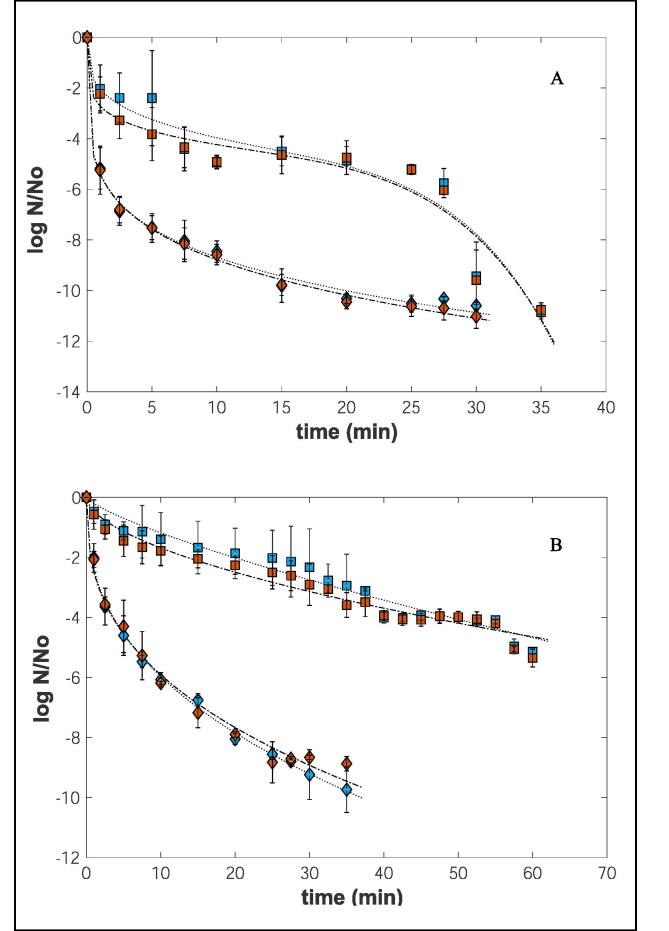
Inactivation curves of *S. aureus* in orange juice during thermosonication (A) at 50 °C (◻) and 60 °C (◇), and during heat treatment (B) at 50 °C (◻) and 60 °C (◇) in two media (Blue: MSA; Red: TSA). The lines … and ._._. represent corresponding models fit to the experimental data. (For interpretation of the references to colour in this figure legend, the reader is referred to the web version of this article.)

As shown in Fig. 4, more than 5 log cycles decay was achieved for both temperatures tested. These outcomes are in line with the FDA requirements for efficient juice processing.

According to Fig. 4A, a decrease of 2.40 ± 1.88, 4.50 ± 0.57, 5.20 ± 0.19 and 10.85 ± 0.27 log cycles was observed in *S. aureus* counts in samples treated with ultrasounds at 50 °C for 5, 15, 25 and 35 min, respectively. This reduction was more accentuated in samples treated at 60 °C, with a decrease of 5.18 ± 0.82 log cycles right after 1 min of treatment.

As can be observed in Fig. 4B, an almost log linear relationship between the number of survivors and treatment time was noted with heat treatment. However, during the thermosonication treatment at 50 °C, a different behavior was observed during inactivation - a non-linear inactivation curve. Although an initial linear reduction was observed from 0 to 10 min, after this period, the cells remained more or less constant for 17.5 min, followed by a fast decrease in microbial survival counts. This period appears to be a crucial phase of cell decline; at the end of the treatment, the cell load reduction was 10 log cycles.

In the case of thermosonication at 60 °C, a non-linear behavior was also visualized. After the log-linear inactivation phase, a long deceleration process at the end of the curve was apparent. This phenomenon is known as tail and is associated with a distribution of intrinsic thermotolerance within the microbial population and the induction of resistance adaptation responses, namely the production of heat shock proteins[21]. The non-linear behavior of *S. aureus* during thermosonication treatment at 50 °C can be explained by (i) the bacteria presence in cluster form; (ii) the action of ultrasound, which has a declumping effect[15], and/or (iii) the protective effect of dead cells or the treatment medium (orange juice pulp). After 25 min of treatment, the microbial clumping could be disrupted, freeing living cells or partially injured cells that would recover by generating the curve’s adapted shape.

A study conducted by Walkling-Ribeiro et al.[9] on *S. aureus* (with an initial concentration of 10^11^ CFU/mL) in treated orange juice with an ultrasonic bath at a frequency of 30 kHz at 55 °C for 5, 10 and 20 min showed linear reductions of 0.8, 1.8 and 3.3 log cycles, respectively. Comparing these results with those obtained in the present study, it can be concluded that thermosonication treatments are more effective using an ultrasonic probe than an ultrasonic bath.

Previous works also found the same magnitude impact of thermosonication treatments in orange juice but using *E. coli* [39]and *Lactobacillus acidophilus*[40]as the target microorganisms. However, microbial sensitivity to thermosonication is influenced by bacterium cell morphology and physiology[41]. It has been shown that, in general, bigger cells would be more sensitive to heat and ultrasonic waves than smaller cells[14]. With the increased surface area, larger cells are subjected to more pressure from ultrasonic cavitation, which leads them to be more sonication vulnerable.

Another factor that needs to be taken into consideration is the juice type. In the current study, the presence of orange pulp has undoubtedly increased the resistance of *S. aureus* to ultrasounds. Similar results were found by Earnshaw et al. [16]that compared the microbial ultrasonic susceptibility in orange juice with and without pulp.

There are many factors in an ultrasound treatment that play an essential role in the microbial inactivation rate: conditions of ultrasound treatment, such as frequency, amplitude, reactor ultrasonic horn orientation, reactor and horn geometry, sampling process and location, acoustic energy density (AED), and the matrix’s complexity. Due to the significant number of relevant parameters, it is crucial to document and report a detailed experimental procedure [42].

Ultrasound is considered a non-thermal technology. Therefore, we can say that temperature is a weakening factor for bacteria during treatment. Still, the destructive element is the cavitation generated by ultrasonic waves that induces the destruction of the cell[21].

As shown in Fig. 4B, heat treatment also affected *S. aureus*’ inactivation. After completing the total treatment time, 5.14 ± 0.08 and 9.74 ± 0.76 log cycles reductions at 50 and 60 °C were achieved, respectively.

From a comparison of Fig. 4A and B, it can be concluded that, for the temperature of 50 °C, the combination of ultrasound and heat reduces the treatment time (from 60 to 35 min) and increases the bacterial inactivation load from 5.14 ± 0.08 to 10.85 ± 0.27 log cycles. When a higher temperature is used (60 °C), the inactivation rate of *S. aureus* is sped up when thermosonication is used. This treatment reduced the initial microbial numbers by 9.81 ± 0.66 log cycles in the first 15 min of treatment compared with the 6.75 ± 0.22 log cycles reductions in heat processing alone.

These results proved that thermosonication processes could increase microbial inactivation rate in orange juice since a synergistic effect was observed between thermal and ultrasound treatments. The mechanisms of microbial inactivation are then due to (i) the temperature increment that leads to a decrease in the juice viscosity enhancing the generation of cavitation bubbles and the subsequent stronger bubble collapse and (ii) higher temperatures that weakens the outer membrane of microbial cells, making the cells less resistant to ultrasound[43].

Previous studies had reported the same synergistic effect [44]. This hurdle allowed a reduction in processing time, leading to a consequent improvement in juice quality[42]. Ugarte-Romero et al. [20] observed that the combination of heat and sonication had better inactivation results of *E. coli* O157:H7 and *Salmonella* *enteritidis* and allowed minimal impact on mango and grapefruit juice quality parameters, compared to ultrasound alone.

It has been suggested that above a given temperature, the rate of microbial inactivation for thermosonication will not increase compared to heat treatment. Based on the results presented in Fig. 4, compared to the heat treatment alone, the thermosonication efficiency is better at 50 °C than at 60 °C. This means that the microbial inactivation will be due to heat rather than to ultrasound-induced cavitation at higher temperatures. Raso et al.[19]also reported that at temperatures above 58 °C, the inactivation effect was only due to heat. This can be explained because, at high temperatures, there is a damping effect and a reduction in cavitation activity, as well as an increase in vapor pressure and a decrease in surface tension[20].

### 3.4 *S. aureus* inactivation modeling

The Weibull model was adequate in all data fits of ultrasonicated, thermal and thermosonicated log-survival data of *S. aureus*, except for the thermosonicated juice at 50 °C. Estimated parameters and coefficient of determination R^2^ are included in Table 2, Table 3 for Weibull and four-parameter/sigmoid models, respectively. The values of R^2^ were very high for all cases, varying from 0.97 to 1.00. However, for heat treatment at 50 °C with MSA, some dispersion of experimental data was observed, and the coefficient of determination decreased to 0.93.

**Table 2:** Estimated parameters for the Weibul model fit to the data. The values are mean ± margin of confidence interval at 95%.

Treatment	k (min^-n^)	n	R^2^
US 20 °C MSA	2.21 ± 0.21	0.13 ± 0.03	0.99
US 20 °C TSA	2.17 ± 0.23	0.14 ± 0.03	0.99
US 30 °C MSA	2.18 ± 0.35	0.14 ± 0.05	0.97
US 30 °C TSA	2.22 ± 0.31	0.14 ± 0.04	0.98
US 40 °C MSA	2.90 ± 0.25	0.09 ± 0.03	0.99
US 40 °C TSA	3.05 ± 0.26	0.09 ± 0.03	0.99
HT 50 °C MSA	0.20 ± 0.09	0.77 ± 0.12	0.93
HT 50 °C TSA	0.46 ± 0.10	0.57 ± 0.06	0.98
HT 60 °C MSA	2.41 ± 0.23	0.39 ± 0.03	1.00
HT 60 °C TSA	2.49 ± 0.27	0.37 ± 0.04	0.99
TS 60 °C MSA	5.43 ± 0.36	0.20 ± 0.02	1.00
TS 60 °C TSA	5.38 ± 0.34	0.21 ± 0.02	1.00

**Table 3:** Estimated parameters for the four-parameter/sigmoid model fit to the data. The values are mean ± margin of confidence interval at 95%.

Treatment	k_1_ (min^-n^1)	n_1_	k_2_ (min^-n^2)	n_2_	R^2^
TS 50 °C MSA	1.64x10^-8^ ± 0.16	5.52 ± 4.23	2.04 ± 0.89	0.29 ± 0.20	0.97
TS 50 °C TSA	9.30x10^-8^ ± 0.91	5.05 ± 2.90	2.72 ± 0.81	0.19 ± 0.15	0.98

The parameter k represents a microbial inactivation rate, or the reciprocal of a first decimal reduction time. As it can be observed in Table 2, k is usually not affected by the platting media. Except for the heat treatment at 50 °C, all the k values were significantly similar between treatments. This variation is probably due to the dispersion of the experimental data above mentioned. For ultrasonicated juices at 20 and 30 °C, k values were not significantly different between them and the ones with heat treatment at 60 °C. This means that the initial velocity of microbial inactivation is similar in the juices ultrasonicated at the lower temperatures and heat treated at the higher temperature studied. According to the data, as temperature increases, k also increases. This means that increasing the temperature of treatments greatly affects the rate of inactivation. Another conclusion is that the inactivation is faster in thermosonicated than in heat treatments since lower k values were obtained for heat treated juices. Other authors also agree that by increasing the temperature, ultrasounds become more powerful and faster for microbial inactivation than heat treatment alone since it enhances the lethal effect of the process[[9], [26], [45]].

In terms of the shape parameter n, all the curves had values lower than 1, indicating an upward concavity of log-survival data. This suggests that the remaining cells have less probability of dying, meaning that the residual cells are more stress adapted. The same results (n < 1) were obtained by Ugarte-Romero et al. [46] for *E. coli* K12 treated by ultrasounds (20 kHz) at 5 different temperatures in apple cider.

However, the survival curve of the thermosonicated juice at 50 °C showed a different shape that could not be fit with the Weibull model. Therefore, a four-parameter model was used to describe the sigmoidal inactivation behaviour. Bermúdez-Aguirre et al.[21]explained this curve as an upward concavity at the beginning due to the inactivation of the weakest cells in the first few minutes of treatment, followed by an almost steady stage. In this period, the cells are more resistant but simultaneously accumulate damage from the heat and ultrasound effects. This leads to a change to a downward concavity resulting in faster inactivation due to increased cell damage.

### 3.5 Energy analysis

**Fig. 5:**
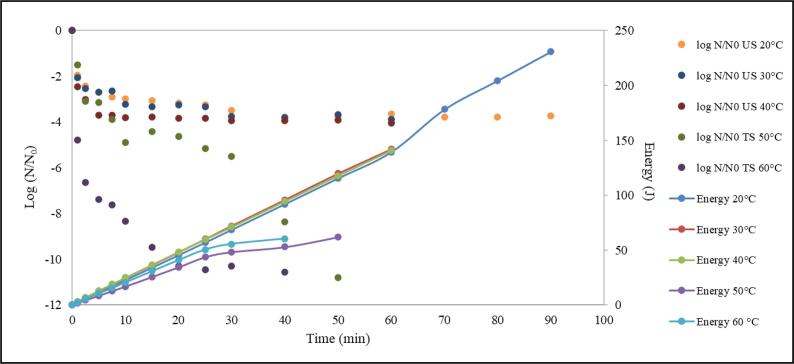
Energy consumed during ultrasonic and thermosonication treatments at 20, 30, 40, 50 and 60 °C.

The ultrasound power source was designed to provide constant amplitudes to the juice sample, regardless of changing conditions. A sensing network continuously monitors the output requirements and automatically adjusts the power to maintain the amplitude at the preselected level. The energy supplied to the probe by the ultrasonic processor at 20, 30, 40, 50 and 60 °C is presented in Fig. 5. As can be observed, the delivered energy increased with the increase of treatment time. As the juice is processed, the probe’s resistance to movement increases due to juice changes (viscosity, concentration, and temperature). Therefore, additional power is required to ensure that the energy remains constant at the probe’s exit.

As also verified in Fig. 5, two different behaviors can be noted depending on the temperature range. When sublethal temperatures were applied (20, 30 and 40 °C) higher amount of energy had to be supplied to the probe to maintain the chosen amplitude. At lethal temperatures (50 and 60 °C), less energy was needed to overcome the probe’s resistance to movement. For the same treatment time (30 min), the energy reached up to 68.70 ± 14.38, 72.19 ± 15.09, 71.16 ± 14.28, 52.88 ± 0.97, and 60.39 ± 2.03 Joules, for ultrasound processes at 20, 30, 40, 50 and 60 °C, respectively.

Studies on the supplied energy during sonication for microbial inactivation are still limited. Gogate [36], and Bermúdez-Aguirre and Barbosa-Cánovas [47] reported the required energy to be applied in different juices for the *Saccharomyces cerevisiae* and *Listeria monocytogenes* inactivation, respectively.

Efforts are made worldwide to provide the maximum possible information in a more standardized and uniform way. Since the energy supplied depends not only on the equipment itself but also on the ultrasonic processing conditions, it is essential to establish standard settings and parameters to compare the results. Besides these structural equipment parameters, the energy required is also significantly influenced by liquid medium properties, such as viscosity, surface tension, and suspended solids concentration.

### 3.6 Effect of thermosonication on *S. aureus* cells morphology through scanning electron microscopy

**Fig. 6:**
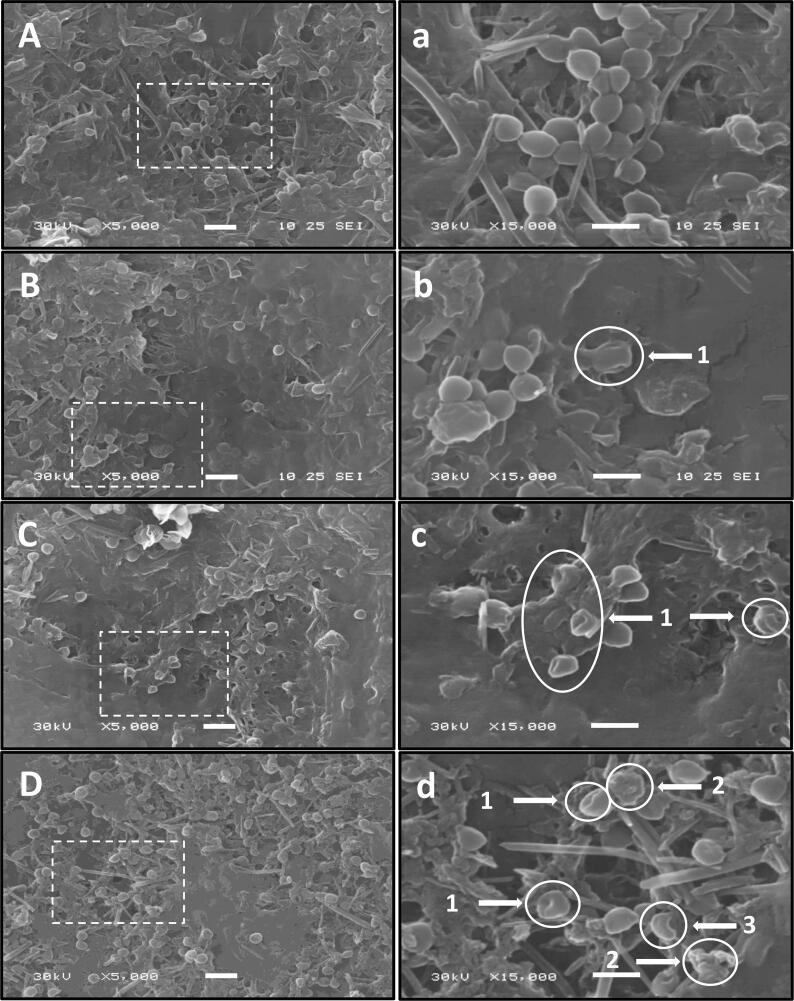
Scanning electron microscope (SEM) images of *S. aureus* treated by ultrasound at different temperatures: untreated orange juice (A: x 5000, a: x 15000), treated at 40 °C for 60 min (B: x 5000, b: x 15000), treated at 50 °C for 35 min (C: x 5000, c: x 15000) and treated at 60 °C for 30 min (D: x 5000, d: x 15000).

The SEM images of untreated *S. aureus* ATCC 29213 (Fig. 6 A, a) displayed intact cells with a characteristic spherical or elliptical shape that occurred in grape-like clusters.

After 60 min of ultrasound treatment at 40 °C, most *S. aureus* clusters were scattered and appeared as single cells. The majority of cells still seemed intact, with only a few showing pore formation and the consequent leakage of some cell contents (Fig. 6 B, b). These results are consistent with those found by [34] on *S. aureus* treated with ultrasounds for 10 min at 20 °C, where they also observed the same cells morphology changes. Although the treatment conditions and the matrix composition on both studies were different, the outcomes indicate that bacterial damages after sublethal ultrasounds can be found mainly due to some membrane damage and consequent leakage of cytoplasm. In another work, it was observed that part of *S. aureus* cells was entirely and irreversibly disrupted after 12 min of ultrasound treatment at 20 °C. These divergent results are probably due to the cells lack of protection by the matrix since, in this previous study, *S. aureus* cells were treated in a bacterial solution. In the present research, the culture was inoculated in orange juice, in which the juice constituents will undoubtedly have given some cells protection.

When ultrasounds were combined with high temperatures (50 and 60 °C), micrographs suggested that *S. aureus* cell contents were strongly extruded from the cells, possibly due to increased intracellular pressure (Fig. 6 C, c and D, d). In samples treated with ultrasound at 50 °C for 35 min, single-pore cells were regularly visualized, from which the cell’s contents are likely to have been ejected (Fig. 6 C, 1). An extensive borderline lesion occurred in *S. aureus* cells exposed to 30 min of sonication at 60 °C. Many cells appeared empty and flattened (Fig. 6 d, 2), in addition to the present fragments of disintegrated cells (Fig. 6 d, 3). This SEM analysis allowed to conclude that ultrasound did not trigger a similar effect on all cells since *S. aureus* cells were unevenly damaged. However, it was evident that when ultrasounds were combined with moderately high temperatures (50 – 60 °C), a significant impact on the morphological appearance of *S. aureus* was noted. According to literature, thermosonication at these temperature ranges would cause a weakening effect on cell envelopes, increasing their susceptibility to cavitation effects [45].

### 3.7 Conclusion

Thermosonication seems to be a viable technology for orange juice pasteurization since it allowed to achieve the 5-log reductions required by FDA regulations.

The most lethal treatment for *S. aureus* ATCC 29213 in this research was at 60 °C for 30 min at 20 kHz, 80% amplitude and using discontinuous mode. At these conditions, 10.60 ± 0.13 log cycles of microbial reductions were attained. Although a similar bacterial reduction was achieved at 50 °C for 30 min (9.44 ± 1.35 log cycles), the inactivation kinetic curves significantly differed. This can represent a threat since, as the microbial behavior is not linear, cases of microbial resistance can be neglected.

The Weibull model was adequate in describing the reduction of microbial loads in almost all the situations analysed, except for the thermosonicated treatment at 50 °C, where the four-parameter model was applied. This modelling approach is an attempt to contribute to the design of more effective processes.

One critical point is to present the energy consumed for the different temperatures of thermosonication treatment, to estimate costs and, in the future, to scale up to the industry level. However, these energy balances must include all sources of heat losses or profits of a perfectly designed system. The energy balance of the non-isolated and open system used for this research showed that orange juice sample required an average of 60.39 ± 2.03 J to reach the required pasteurization standards.

As observed, using electron microscope analysis, the synergistic effect between ultrasounds and heat might be summarized on the disaggregation impact of ultrasound that dispersed *S. aureus* clusters into single cells, which increased the probabilities of interaction with heat. Damaged *S. aureus* cells were further ruptured by ultrasound, which caused cells lyses with fragmentized walls and membranes. These results indicated that the pasteurization technique of combined ultrasound and heat was an environmentally friendly, cost-effective and easy-to-perform approach with a high-efficiency degree.

## 4 Data availability

Data will be available on reasonable request.

## CRediT authorship contribution statement

**Akila Amir Tahi:** Investigation, Methodology, Validation, Data curation, Writing - original draft. **Sérgio Sousa:** Methodology, Writing - original draft. **Khodir Madani:** Project administration, Supervision. **Cristina L.M. Silva:** Conceptualization, Resources, Validation, Formal analysis, Investigation, Supervision, Writing - review & editing. **Fátima A. Miller:** Conceptualization, Validation, Formal analysis, Investigation, Data curation, Writing - original draft, Supervision, Writing - review & editing.

## Declaration of Competing Interest

The authors declare that they have no known competing financial interests or personal relationships that could have appeared to influence the work reported in this paper.

## References

[1] Grace D. (2015). Food safety in low and middle income countries. Int. J. Environ. Res. Public Health.

[2] Grace D., Fetsch A. (2018).

[3] Li J., Ahn J., Liu D., Chen S., Ye X., Ding T., Elkins C.A. (2016). Evaluation of ultrasound-induced damage to Escherichia coli and *Staphylococcus aureus* by flow cytometry and transmission electron microscopy. Appl. Environ. Microbiol..

[4] Bhatia A., Zahoor S. (2007). *Staphylococcus aureus* enterotoxins: a review. J Clin Diag Res.

[5] Hennekinne J.-A., De Buyser M.-L., Dragacci S. (2012). *Staphylococcus aureus* and its food poisoning toxins: characterization and outbreak investigation. FEMS Microbiol. Rev..

[6] Schmitt M., Schuler-Schmid U., Schmidt-Lorenz W. (1990). Temperature limits of growth, TNase and enterotoxin production of *Staphylococcus aureus* strains isolated from foods. Int. J. Food Microbiol..

[7] Abid M., Jabbar S., Wu T., Hashim M.M., Hu B., Lei S., Zhang X., Zeng X. (2013). Effect of ultrasound on different quality parameters of apple juice. Ultrason. Sonochem..

[8] Salleh-Mack S.Z., Roberts J.S. (2007). Ultrasound pasteurization: the effects of temperature, soluble solids, organic acids and pH on the inactivation of Escherichia coli ATCC 25922. Ultrason. Sonochem..

[9] Walkling-Ribeiro M., Noci F., Riener J., Cronin D.A., Lyng J.G., Morgan D.J. (2009). The impact of thermosonication and pulsed electric fields on *Staphylococcus aureus* inactivation and selected quality parameters in orange juice. Food Bioprocess Technol..

[10] Chemat F., Zill-e-Huma, Khan M.K. (2011). Applications of ultrasound in food technology: processing, preservation and extraction. Ultrason. Sonochem..

[11] Koda S., Miyamoto M., Toma M., Matsuoka T., Maebayashi M. (2009). Inactivation of Escherichia coli and Streptococcus mutans by ultrasound at 500 kHz. Ultrason. Sonochem..

[12] Majid I., Nayik G.A., Nanda V., Yildiz F. (2015). Ultrasonication and food technology: A review. Cogent Food & Agriculture.

[13] Gogate P.R., Kabadi A.M. (2009). A review of applications of cavitation in biochemical engineering/biotechnology. Biochem. Eng. J..

[14] Yusaf T., Al-Juboori R.A. (2014). Alternative methods of microorganism disruption for agricultural applications. Appl. Energy.

[15] Joyce E., Phull S., Lorimer J., Mason T. (2003). The development and evaluation of ultrasound for the treatment of bacterial suspensions. A study of frequency, power and sonication time on cultured Bacillus species. Ultrason. Sonochem..

[16] Earnshaw R.G., Appleyard J., Hurst R.M. (1995). Understanding physical inactivation processes: Combined preservation opportunities using heat, ultrasound and pressure. Int. J. Food Microbiol..

[17] López-Malo A., Guerrero S., Alzamora S. (1999). Saccharomyces cerevisiae thermal inactivation kinetics combined with ultrasound. J. Food Prot..

[18] Ordoñez J.A., Aguilera M.A., Garcia M.L., Sanz B. (1987). Effect of combined ultrasonic and heat treatment (thermoultrasonication) on the survival of a strain of *Staphylococcus aureus*. J. Dairy Res..

[19] Raso J., Pagán R., Condón S., Sala F.J. (1998). Influence of temperature and pressure on the lethality of ultrasound. Appl. Environ. Microbiol..

[20] Ugarte-Romero E., Feng H., Martin S.E. (2007). Inactivation of Shigella boydii 18 IDPH and Listeria monocytogenes Scott A with power ultrasound at different acoustic energy densities and temperatures. J. Food Sci..

[21] Bermúdez-Aguirre D., Corradini M.G., Mawson R., Barbosa-Cánovas G.V. (2009). Modeling the inactivation of Listeria innocua in raw whole milk treated under thermo-sonication. Innovative Food Sci. Emerg. Technol..

[22] Monod J. (1949). The growth of bacterial cultures. Annu. Rev. Microbiol..

[23] Novick A. (1955). Growth of bacteria. Annual Reviews in Microbiology.

[24] Gao S., Lewis G.D., Ashokkumar M., Hemar Y. (2014). Inactivation of microorganisms by low-frequency high-power ultrasound: 1. Effect of growth phase and capsule properties of the bacteria. Ultrason. Sonochem..

[25] Belay N., Rasooly A. (2002). *Staphylococcus aureus* growth and enterotoxin A production in an anaerobic environment. J. Food Prot..

[26] Abedi-Moghaddam N., Bulic A., Herderson L., Lam E. (2004). Survival of Escherichia coli to UV irradiation during exponential and stationary phases of growth. J. Exp. Microbiol. Immunol.

[27] Twentyman P.R., Bleehen N.M. (1973). The sensitivity of cells in exponential and stationary phases of growth to bleomycin and to 1, 3-bis (2-chloroethyl)-1-nitrosourea. Br. J. Cancer.

[28] Azizkhani M., Misaghi A., Basti A.A., Gandomi H., Hosseini H. (2013). Effects of Zataria multiflora Boiss. essential oil on growth and gene expression of enterotoxins A, C and E in *Staphylococcus aureus* ATCC 29213. Int. J. Food Microbiol..

[29] Schleifer, K.-H. (2009). Phylum XIII. Firmicutes Gibbons and Murray 1978, 5 (Firmacutes [sic] Gibbons and Murray 1978, 5). In Bergey’s Manual® of Systematic Bacteriology (pp. 19-1317): Springer.

[30] Todar, K. (2004). Todar's online textbook of bacteriology. In: University of Wisconsin Madison^ eWisconsin Wisconsin.

[31] Li J., Ding T., Liao X., Chen S., Ye X., Liu D. (2017). Synergetic effects of ultrasound and slightly acidic electrolyzed water against *Staphylococcus aureus* evaluated by flow cytometry and electron microscopy. Ultrason. Sonochem..

[32] Clarke P.R., Hill C.R. (1970). Physical and chemical aspects of ultrasonic disruption of cells. The Journal of the Acoustical Society of America.

[33] Russell N.J., Colley M., Simpson R.K., Trivett A.J., Evans R.I. (2000). Mechanism of action of pulsed high electric field (PHEF) on the membranes of food-poisoning bacteria is an ‘all-or-nothing’effect. Int. J. Food Microbiol..

[34] Li J., Suo Y., Liao X., Ahn J., Liu D., Chen S., Ye X., Ding T. (2017). Analysis of *Staphylococcus aureus* cell viability, sublethal injury and death induced by synergistic combination of ultrasound and mild heat. Ultrason. Sonochem..

[35] Evelyn S., F. V. M. (2020). Ultrasound assisted thermal inactivation of spores in foods: Pathogenic and spoilage bacteria, molds and yeasts. Trends Food Sci. Technol..

[36] Gogate P.R. (2007). Application of cavitational reactors for water disinfection: current status and path forward. J. Environ. Manage..

[37] Monsen T., Lövgren E., Widerström M., Wallinder L. (2009). In vitro effect of ultrasound on bacteria and suggested protocol for sonication and diagnosis of prosthetic infections. J. Clin. Microbiol..

[38] Drakopoulou S., Terzakis S., Fountoulakis M.S., Mantzavinos D., Manios T. (2009). Ultrasound-induced inactivation of gram-negative and gram-positive bacteria in secondary treated municipal wastewater. Ultrason. Sonochem..

[39] Utsunomiya, Y., & Kosaka, Y. (1979). Application of supersonic waves to foods. Journal of the Faculty of Applied Biological Science Hiroshima University.

[40] Zenker M., Heinz V., Knorr D. (2003). Application of ultrasound-assisted thermal processing for preservation and quality retention of liquid foods. J. Food Prot..

[41] Food U., Administration D. (2006). Procedures for the safe and sanitary processing and importing of juice.

[42] Lee H., Zhou B., Liang W., Feng H., Martin S.E. (2009). Inactivation of Escherichia coli cells with sonication, manosonication, thermosonication, and manothermosonication: microbial responses and kinetics modeling. J. Food Eng..

[43] Baboli Z.M., Williams L., Chen G. (2020). Design of a batch ultrasonic reactor for rapid pasteurization of juices. J. Food Eng..

[44] López-Malo A., Palou E., Jiménez-Fernández M., Alzamora S.M., Guerrero S. (2005). Multifactorial fungal inactivation combining thermosonication and antimicrobials. J. Food Eng..

[45] Álvarez I., Mañas P., Sala F.J., Condón S. (2003). Inactivation of Salmonella enterica serovar Enteritidis by ultrasonic waves under pressure at different water activities. Appl. Environ. Microbiol..

[46] Ugarte-Romero E., Feng H., Martin S.E., Cadwallader K.R., Robinson S.J. (2006). Inactivation of Escherichia coli with power ultrasound in apple cider. J. Food Sci..

[47] Bermúdez-Aguirre D., Barbosa-Cánovas G.V. (2012). Inactivation of Saccharomyces cerevisiae in pineapple, grape and cranberry juices under pulsed and continuous thermo-sonication treatments. J. Food Eng..

